# Contribution of LTi and T_H_17 cells to B cell aggregate formation in the central nervous system in a mouse model of multiple sclerosis

**DOI:** 10.1186/s12974-019-1500-x

**Published:** 2019-05-28

**Authors:** Verena Schropp, Jörn Rohde, Damiano M. Rovituso, Samir Jabari, Richa Bharti, Stefanie Kuerten

**Affiliations:** 1 Institute of Anatomy, Chair of Anatomy and Cell Biology, Friedrich-Alexander University Erlangen-Nürnberg (FAU), Erlangen, Germany; 2 Institute of Anatomy and Cell Biology, University of Würzburg, Würzburg, Germany; 3 Core Unit Systems Medicine, University Hospitals of Würzburg, Würzburg, Germany

**Keywords:** EAE, LTi cells, MP4, MS, T_H_17 cells

## Abstract

**Background:**

In a subgroup of patients suffering from progressive multiple sclerosis (MS), which is an inflammation-mediated neurodegenerative disease of the central nervous system (CNS), B cell aggregates were discovered within the meninges. Occurrence of these structures was associated with a more severe disease course and cortical histopathology. We have developed the B cell-dependent MP4-induced experimental autoimmune encephalomyelitis (EAE) as a mouse model to mimic this trait of the human disease. The aim of this study was to determine a potential role of lymphoid tissue inducer (LTi) and T_H_17 cells in the process of B cell aggregate formation in the MP4 model.

**Methods:**

We performed flow cytometry of cerebellar and splenic tissue of MP4-immunized mice in the acute and chronic stage of the disease to analyze the presence of CD3^−^CD5^−^CD4^+^RORγt^+^ LTi and CD3^+^CD5^+^CD4^+^RORγt^+^ T_H_17 cells. Myelin oligodendrocyte glycoprotein (MOG):35–55-induced EAE was used as B cell-independent control model. We further determined the gene expression profile of B cell aggregates using laser capture microdissection, followed by RNA sequencing.

**Results:**

While we were able to detect LTi cells in the embryonic spleen and adult intestine, which served as positive controls, there was no evidence for the existence of such a population in acute or chronic EAE in neither of the two models. Yet, we detected CD3^−^CD5^−^CD4^−^RORγt^+^ innate lymphoid cells (ILCs) and T_H_17 cells in the CNS, the latter especially in the chronic stage of MP4-induced EAE. Moreover, we observed a unique gene signature in CNS B cell aggregates compared to draining lymph nodes of MP4-immunized mice and to cerebellum as well as draining lymph nodes of mice with MOG:35–55-induced EAE.

**Conclusion:**

The absence of LTi cells in the cerebellum suggests that other cells might take over the function as an initiator of lymphoid tissue formation in the CNS. Overall, the development of ectopic lymphoid organs is a complex process based on an interplay between several molecules and signals. Here, we propose some potential candidates, which might be involved in the formation of B cell aggregates in the CNS of MP4-immunized mice.

## Background

To initiate adaptive immune responses and regulate immune processes, highly complex structures, called secondary lymphoid organs (SLOs), are formed during embryogenesis. While SLOs are anatomically distinct tissues and involved in acute inflammatory reactions, ectopic lymphoid structures can evolve when inflammation persists for a long time [1]. The development of these so-called tertiary lymphoid organs (TLOs) has been mainly described to occur during the course of autoimmune processes [2]. Beside in autoimmune diseases like rheumatoid arthritis and myasthenia gravis [2], ectopic lymphoid structures have also been identified in a subgroup of patients suffering from progressive multiple sclerosis (MS) [3]. By analyzing postmortem brain tissue, B cell follicle-like structures were discovered in the meninges of these patients [3]. Occurrence of such structures was associated with a more severe disease course and cortical histopathology [4]. These results suggest that the presence of ectopic lymphoid tissue could play an important role in the pathophysiology of MS. Indeed, TLOs may provide suitable conditions for immune cells to undergo maturation, to interact with each other, and to subsequently trigger immune reactions, which aggravate the disease course [5]. To further investigate the role of TLOs in MS and to get deeper insights into the formation of these structures we used experimental autoimmune encephalomyelitis (EAE), which is the most common mouse model of MS. We have previously established MP4-induced EAE, which is induced by a fusion protein consisting of the human isoform of myelin basic protein (MBP) and the three hydrophilic domains of proteolipid protein (PLP) [6]. Immunization with MP4 triggers the activation of both antigen-specific T cells and B cells, which can be detected in the blood, along with MP4-specific IgG [6–9]. Similar to other EAE models, MP4-immunized mice show immune cell infiltration into the central nervous system (CNS), including both the brain and spinal cord [10]. Further studies have shown a change in the composition of infiltrating cells during the course of disease [11]. In contrast to the presence of CD4^+^ T cells, macrophages, and granulocytes in the acute stage of the disease, chronic EAE mice displayed a predominance of B cells, CD8^+^ T cells, and dendritic cells. Moreover, we have demonstrated that B cell-mediated processes take place in MP4-immunized mice. On the one hand, B cell aggregates were observed mainly in the cerebellum, which increasingly re-organized into TLOs with compartmentalized B cell and T cell zones and high endothelial venules (HEVs) [12]. In addition, we have reported that CD10 is expressed by B cells within the aggregates [13], which is thought to be a relevant marker for B cell differentiation in follicular centers [14]. Furthermore, we have provided evidence of antibody isotype switching in the CNS of MP4-immunized mice [13]. We also detected different clonotypes in the cerebellum compared to the spleen, and we observed antibody epitope spreading [13]. Because of its B cell independence and the absence of B cell aggregates, we also used myelin oligodendrocyte glycoprotein (MOG):35–55-immunized mice in the present study as a control model [11, 15]. Comparing the structure and morphology of TLOs and SLOs, it becomes obvious that ectopic lymphoid tissue resembles SLOs in many aspects. In addition to a similar vascular system, the cells and chemokines observed in TLOs are comparable to those in SLOs [16]. During embryogenesis, lymphoid tissue inducer cells (LTi) are considered as the initiators of lymphoid organ formation [17, 18]. These cells belong to the family of innate lymphoid cells (ILCs), which are part of the innate immune system [19]. LTi cells derive from the liver and migrate to induce the formation of SLOs by lymphotoxin signaling, thereby stimulating stroma cells [2, 20, 21]. The expression of chemokines and adhesion molecules causes the recruitment of different cells and finally the organization of the complex structures [2, 21]. Whether LTi cells also contribute to the development of ectopic lymphoid tissue in the brain of mice and eventually of MS patients needs to be investigated. Previous research has demonstrated that LTi cells can induce the formation of lymphoid tissue in the periphery of mice, e.g., new Peyer’s patches [22, 23]. Recently, Serafini and colleagues detected a small amount of CD3^–^RORγt^+^ cells, potentially ILC3/LTi cells, in B cell aggregates/follicles and adjacent diffuse meningeal infiltrates in secondary progressive MS patients [24]. Another cell population, which has also been associated with ectopic lymphoid tissue formation, is T_H_17 cells. For instance, MOG:35–55-specific T_H_17 were shown to induce the formation of ectopic lymphoid tissue in the CNS of mice after passive transfer into C57BL/6 (B6) recipient mice [25]. Apparently, the induction relied on a close interaction between T_H_17 cells, meningeal fibroblastic reticular cells, and the secretion of CXCL13, which in turn was dependent on lymphotoxin and necessary for B cell aggregation [26]. Interestingly, the comparison of the expression profile of T_H_17 and LTi cells suggests striking similarity between the two cell types. Markers like the transcription factor RORγt in addition to CD4, interleukin (IL)-7R, IL-22, or IL-17 have been reported in both cell populations [19, 27]. One focus of our study was to investigate the role of LTi and T_H_17 cells in ectopic lymphoid tissue formation in the CNS of MP4-immunized mice. Besides these two cell populations, it is conceivable that several other factors are necessary to support the highly complex structure of a TLO. To determine potential other candidate molecules relevant to TLO formation and maintenance, we also studied the gene expression profile of B cell aggregates.

## Materials and methods

### Mice

Six-week-old female B6 mice were purchased from Janvier (France) and maintained at the animal facility of the Zentrum für Mund- und Kiefergesundheit at the University of Würzburg under specific pathogen-free conditions. Mice were fed with a standard rodent diet (Altromin Spezialfutter GmbH & Co. KG, Lage, Germany) and autoclaved water. Food and water were kept at ground level for mice displaying paralytic symptoms. Pregnant mice were obtained from the Institute of Virology of the University of Würzburg to analyze the embryonic stage of murine spleen development. A total of 69 mice was used in our study. All animal experiments were approved by the Regierung von Unterfranken (approval number 91/14) and were in accordance with the German Law on the Protection of Animals, the “Principles of laboratory animal care” (NIH publication no. 86–23, revised 1985) and the ARRIVE (Animal Research: Reporting of In Vivo Experiments) guidelines.

### EAE induction and clinical assessment

**Fig. 1:**
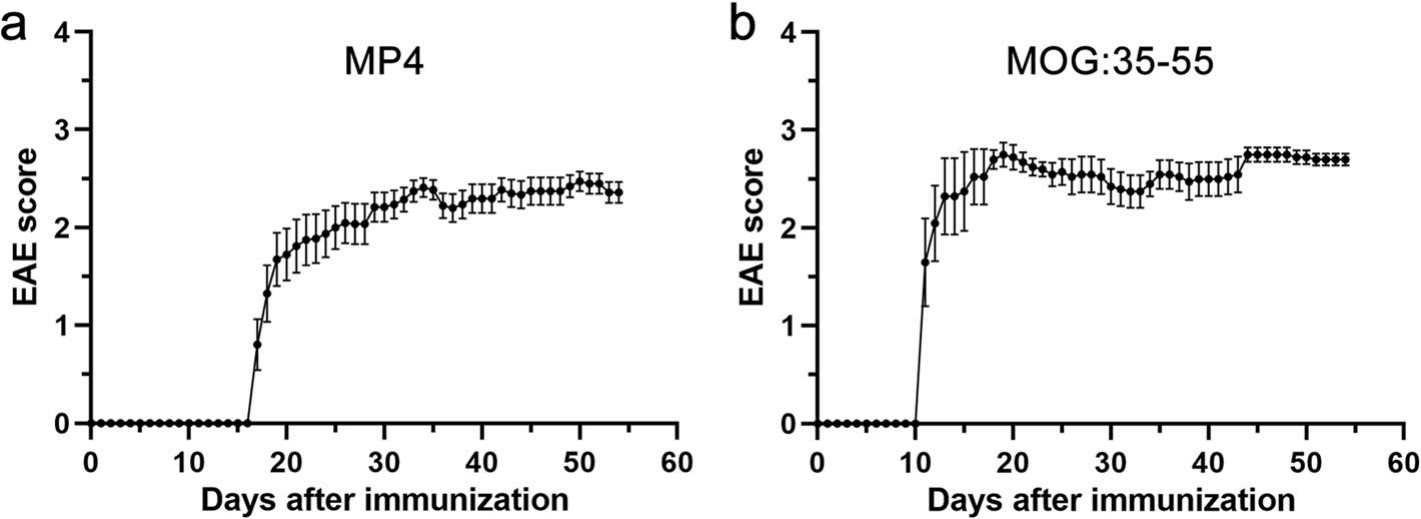
Clinical disease course of **a** MP4 (*n* = 20)- and **b** MOG:35–55 (*n* = 10)-induced EAE, shown until day 54 after immunization. The EAE score was assessed daily. Mean values ± SEM are given

For immunization, incomplete Freund’s adjuvant (IFA) was prepared by mixing paraffin oil (Sigma-Aldrich, St. Louis, USA; Cat # 18512) and mannide monooleate (Sigma-Aldrich; Cat # M8819) at a 9:1 ratio. Complete Freund’s adjuvant (CFA) was subsequently obtained by adding 5 mg/ml *Mycobacterium tuberculosis* H37 Ra (Difco Laboratories, Franklin Lakes, NJ, USA; Cat # 231141) to IFA. After emulsifying MP4 (Alexion Pharmaceuticals, Cheshire, CT, USA) in CFA, the mice were immunized subcutaneously into both sides of the flank with a total dose of 200 μg MP4. Additionally, an intraperitoneal injection of 200 ng pertussis toxin (List Biological Laboratories, Hornby, ONT, Canada; Cat # 181) was given at the day of immunization and 48 h later. For control purposes, mice were immunized with MOG:35–55 (AnaSpec Inc., Fremont, CA, USA; Cat # AS-60130-1) emulsified in CFA at a total dose of 100 μg per mouse. Clinical assessment of EAE was performed daily according to the standard EAE scoring system (Table 1): (0) no disease, (1) floppy tail, (2) hind limb weakness, (3) full hind limb paralysis, (4) quadriplegia, and (5) death. Mice which were in between the defined gradations of the scale were scored in increments of 0.25. Our protocol required mice with a clinical disease score greater than 3 to be culled. However, none of the animals used for the experiments presented here fulfilled this criterion. The disease course of both models is shown in Fig. 1.

**Table 1:** Clinical disease parameters of EAE

	EAE onset (days after immunization)	Time point of experiment	Final EAE score
Mice used for flow cytometry			
MP4-immunized mice at acute stage of disease			
*n* = 2 × 5	14.40 ± 0.37	16.00 ± 0.33	2.55 ± 0.20
MP4-immunized mice at chronic stage of disease			
*n* = 1 × 5 and 1 × 6	18.18 ± 0.52	72.10 ± 0.31	2.48 ± 0.08
MOG:35–55-immunized mice at acute stage of disease			
*n* = 2 × 5	11.90 ± 0.59	13.50 ± 0.50	2.58 ± 0.20
MOG:35–55-immunized mice at chronic stage of disease			
*n* = 2 × 5	12.40 ± 0.79	54.00 ± 0.00	2.70 ± 0.06
Mice used for RNA sequencing			
MP4-immunized mice at chronic stage of disease			
*n* = 9	21.00 ± 1.59	58.44 ± 1.76	2.22 ± 0.22
MOG:35–55-immunized mice at acute stage of disease			
*n* = 5	10.60 ± 0.40	13.00 ± 0.00	2.70 ± 0.05

The mean of the values ± SEM is shown

### Tissue sampling and cell isolation for flow cytometry analysis

#### Spleen and cerebellum

After culling the mice with CO_2_, the cerebellum and spleen were dissected. Cells were isolated from the spleen using 70-μm cell strainers. Cells were incubated in lysis buffer for 10 min on ice to get rid of red blood cells. Density gradient centrifugation was performed for separating cells from the cerebellum. To this end, the cerebellum was homogenized in 1× HBSS^+/+^ (Thermo Fisher Scientific, Waltham, MA, USA; Cat # 14025-050). Thereupon, the stock isotonic Percoll consisting of Percoll^TM^ Plus (GE Healthcare Bio-Sciences AB, Uppsala, Sweden; Cat # 17-5445-02) and 10× HBSS^−**/**−^ (Thermo Fisher Scientific; Cat # 14185-045) was prepared. By mixing the cell suspension with the stock a 30% solution was obtained. To prepare a 70% solution, the stock isotonic Percoll was diluted with 1× HBSS^−/−^ (Thermo Fisher Scientific; Cat # 14185-045). The 30% solution was slowly pipetted onto the 70% solution to set up a density gradient and afterwards centrifuged at 500×*g* at 18 °C for 30 min without break. After the isolation of cells from the interlayer, 1× HBSS^+/+^ (Thermo Fisher Scientific) was used for washing and the cells were resuspended in phosphate-buffered saline (PBS). Cells of both kinds of tissue were equally processed according to the fluorescence-activated cell sorting (FACS) surface and intracellular staining procedure.

#### Intestine

First, the extraction medium was prepared by mixing RPMI medium (Thermo Fisher Scientific; Cat # 11875-093), EDTA, and fetal bovine serum (FBS; GE Healthcare Life Sciences, South Logan, UT, USA; Cat # SV30160.03). For the digestion solution, FBS was added to RPMI medium. Mice were culled with CO_2_ and the small intestine was dissected. Subsequently, the tissue was kept in cold RPMI, containing 10% FBS. Fat was removed from the small intestine, and a syringe with cold PBS was used to get rid of the excrements. After cutting the small intestine into segments and removing the residual fat, the intestinal segments were inverted from the inside to the outside. Before using extraction medium, dithiothreitol (DDT; Thermo Fisher Scientific; Cat # R0861) was added to this solution. The tissue was stirred in the extraction medium at 500 rpm and 37 °C for 15 min. Afterwards, the medium was strained to separate tissue from the solution. The segments were washed in RPMI and the residual mucus was removed by using a dry paper towel. The digestion solution was mixed with dispase (Thermo Fisher Scientific; Cat # 17105041) and collagenase II (Worthington Biochemical Corporation, Lakewood, NJ, USA; Cat # CLS-2) and the tissue was homogenized in a small amount of this medium. Subsequently, this suspension and the residual digestion medium were combined and stirred at 500 rpm and 37 °C for 15 min. After pipetting the suspension up and down, the stirring process was repeated. The digested intestine was filtered through a 70-μm strainer and before centrifugation at 500×*g* at 4 °C for 10 min, RPMI containing 10% FBS was added. The pellet was resuspended and a further filtering step was performed by using a 40-μm cell strainer. The suspension was centrifuged again at the same conditions. The pellet was resuspended in cold PBS and stained according to the protocol described below.

#### Embryonic spleen assay

Pregnant mice were culled at E 15 using CO_2_. After dissecting the uterus, embryos were removed from the uterus horns. The embryos were kept on ice in PBS and heads were cut off. The preparation of the spleen was performed by using a microscope and forceps. The following process was performed according to the previous protocol for the isolation of adult spleen cells.

### Staining of surface markers

Cell suspensions were incubated with BD Horizon™ Fixable Viability Stain 450 (BD Biosciences, San Jose, CA, USA; Cat # 562247) at 4 °C for 30 min in the dark except the intestinal tissue and washed with ice-cold PBS. For the intestine, Fixable Viability Stain 520 (BD Biosciences; Cat # 564407) was used at 4 °C for 15 min in the dark. After adding anti-mouse CD16/CD32 (Thermo Fisher Scientific; Cat # 14-0161-85) to the samples, they were kept for 20 min in the fridge followed by a washing step. Subsequently, cells were stained with fluorochrome-conjugated anti-mouse antibodies at 4 °C for 30 min in the dark. Afterwards, FACS Flow^TM^ (BD Biosciences; Cat # 342003) was added for washing.

### Intracellular staining

Foxp3 fixation/permeabilization working solution was prepared consisting of one part of Foxp3 fixation/permeabilization concentrate (Thermo Fisher Scientific; Cat # 00-5123-43) and three parts of fixation/permeabilization diluent (Thermo Fisher Scientific; Cat # 00-5223-56). Additionally, 10× permeabilization buffer (Thermo Fisher Scientific; Cat # 00-8333-56) was diluted with distilled water to obtain a 1× buffer. After adding the working solution to the cells, they were incubated at room temperature in the dark for 45 min and washed by using the 1× permeabilization buffer. Subsequently, mouse serum (Sigma-Aldrich; Cat # M5905) was pipetted onto the samples. After an incubation time of 15 min at room temperature, fluorochrome-labeled antibodies were added to the cells, which were then kept protected from light at room temperature for 30 min. Finally, the cells were washed with 1× permeabilization buffer and FACSFlow^TM^ (BD Biosciences). For flow cytometry analysis the samples were resuspended in FACSFlow^TM^ (BD Biosciences).

### Flow cytometry analysis

#### Cerebellum, adult and embryonic spleen, intestine

**Fig. 2:**
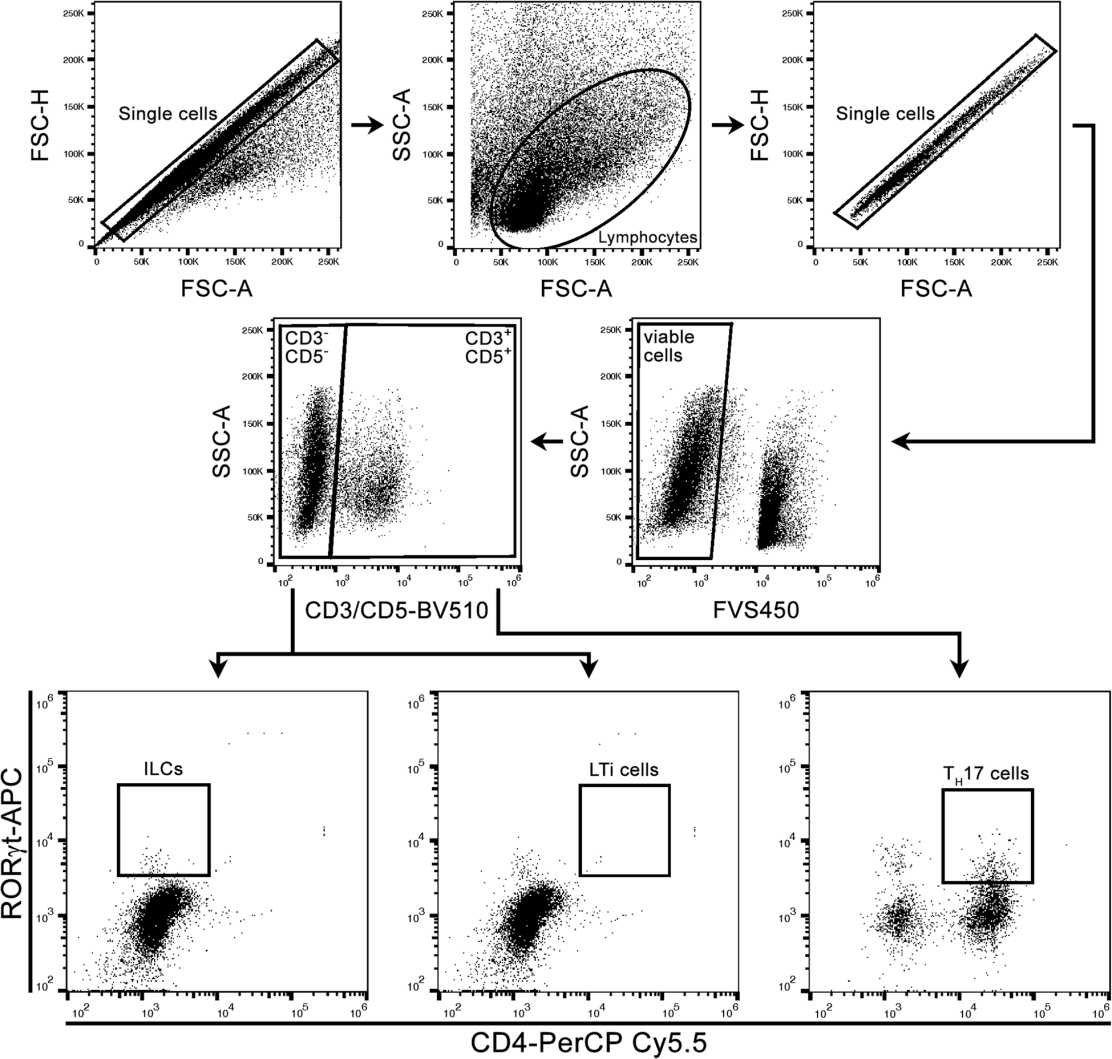
Flow cytometry gating strategy for identification of ILCs, LTi, and T_H_17 cells in EAE mice. Forward scatter area (FSC-A) and height (FSC-H) were used to exclude cells, which had formed doublets. The lymphocyte gate was determined by using FSC-A and sideward scatter area (SSC-A). Viable cells were separated from dead cells. Cells were divided into CD3^−^CD5^−^ and CD3^+^CD5^+^ cells. CD3^−^CD5^−^ cells were further analyzed for the presence of LTi cells and ILCs, and CD3^+^CD5^+^ cells were further analyzed for T_H_17 cells. CD3^−^CD5^−^ cells being positive for CD4 and RORγt represented CD3^−^CD5^−^CD4^+^RORγt^+^ LTi cells. Moreover, CD3^−^CD5^−^CD4^−^RORγt^+^ cells appeared in the ILC gate. CD3^+^CD5^+^ cells, which were also positive for CD4 and RORγt, occurred in the gate of T_H_17 cells

All samples were measured on a FACS Canto^TM^ II flow cytometer (BD Biosciences). The following fluorochrome-conjugated anti-mouse antibodies were selected for identification of ILCs, LTi, and T_H_17 cells: anti-CD4, anti-CD3ε, and anti-CD5 for surface staining and anti-RORγt for intracellular staining (Table 2). To characterize LTi cells in the small intestine, we additionally used anti-mouse CD127 antibody. The analysis of the data was performed with FlowJo software (version 10.07 for Windows, Tree Star, Ashland, OR, USA). The following gating strategy was applied to separate ILC, LTi, and T_H_17 cells (Fig. 2). Undesirable cell populations and doublets were eliminated by using cell size (forward scatter (FSC)) and the granularity (sideward scatter (SSC)). After exclusion of dead cells, surface markers and the intracellular marker were used to characterize the cells. To differentiate between LTis and T_H_17 cells, cells were divided into CD3^−^CD5^−^ cells and CD3^+^CD5^+^ cells. Former studies have described a T cell contamination in the ILC gate when only using CD3 to separate T cells from ILCs. Accordingly, the additional use of CD5 was suggested to exclude undesirable T cells [28]. Subsequently, two-parameter density plots, with CD4 on the *x*-axis and RORγt on the *y*-axis, were used to finally identify CD3^−^CD5^−^CD4^+^RORγt^+^ LTi cells and CD3^+^CD5^+^CD4^+^RORγt^+^ T_H_17 cells. Additionally, CD3^−^CD5^−^CD4^−^RORγt^+^ ILCs were determined by following the first steps of the LTi gating strategy. To distinguish between LTi cells and other members of the third group of ILCs, we have included CD4 as an additional marker [19].

**Table 2:** Antibodies used for flow cytometry

Antibody	Host species	Fluorochrome	Clone	Catalog no.	Origin
Anti-mouse CD3ε	Hamster	Brilliant Violet 510^TM^	145-2C11	100353	BioLegend, San Diego, CA, USA
Anti-mouse CD4	Rat	PerCP-Cy™ 5.5	RM4-5	561115	BD Biosciences, San Jose, CA, USA
Anti-mouse CD5	Rat	Brilliant Violet 510^TM^	53-7.3	100627	BioLegend, San Diego, CA, USA
Anti-mouse CD127	Rat	BV421	SB/199	562959	BD Biosciences, San Jose, CA, USA
Anti-mouse RORγt	Rat	APC	B2D	17-6981-82	Thermo Fisher Scientific, Waltham, MA, USA

### Tissue sampling and cryosectioning

The following procedures for gene sequencing analysis were performed under RNAse-free conditions. Mice were culled with CO_2_. After the dissection of the cerebellum and lymph nodes, the tissue was embedded in Tissue-Tek® (Sakura, Torrance, CA, USA; Cat # 4583) and frozen in liquid nitrogen. Until the continuation of the experiments, samples were preserved at − 80 °C. The frozen tissue was cut into 10-μm-thick sections using a Leica CM3050 S cryostat. For laser capture microdissection (LCM), samples were placed on FrameSlides (Leica; MicroDissect GmbH, Herborn, Germany; Cat # 11505190), which are special microscope slides, consisting of a polyethylene terephthalate (PET) membrane and a steel frame. Furthermore, SuperFrost® Plus slides (Thermo Fisher Scientific; Cat # J1810AMNT) were used for every fifth section to perform immunohistochemical analysis. All slides were stored at − 80 °C until further analysis.

### Immunohistochemical analysis

Cryosections were dried at room temperature for 2 h. During the following entire procedure, washing steps were performed in between. The sections were fixed with 4% paraformaldehyde (PFA) at room temperature for 10 min, protected from light. To inhibit endogenous peroxidase activity, the tissue was exposed to aqueous 3% hydrogen peroxide (H_2_O_2_) solution for 10 min at room temperature in the dark. Afterwards, 5% normal goat serum (NGS; Sigma-Aldrich; Cat # G9023) in PBS was pipetted onto every slide for 1 h incubation to block unspecific binding. Subsequently, samples apart from control slides were incubated overnight at 4 °C protected from light with primary rat anti-mouse B220 antibody (Thermo Fisher Scientific; Cat # 14-0452-81; 1:1000 dilution). The next day, goat anti-rat IgG antibody (Vector Laboratories; Maraval LifeSciences, Burlingame, CA, USA; Cat # BA-9400; 1:500 dilution) was added. After an incubation time of 1 h in the dark at room temperature, samples were exposed to avidin-biotin complex (Vectastain® ABC Kit, Peroxidase Standard, Vector Laboratories; Cat # PK-4000) at room temperature in the dark for 30 min. To start the staining reaction, DAB Peroxidase Substrate Kit (Vector Laboratories; Cat # SK-4100) was pipetted on the tissue and the process was monitored by using a Zeiss Primo Star light microscope until a dark brown reaction product was visible. For stopping the staining process, PBS was used. Before dehydrating samples in ethanol and xylene baths, the tissue was stained with 0.1% Kernechtrot (Merck, Darmstadt, Germany; Cat # 5189) dissolved in 5% aluminiumsulfate-18-hydrate (Sigma-Aldrich; Cat # 11044) solution. Finally, the samples were mounted in DePeX (Serva, Heidelberg, Germany; Cat # 18243.02). For additional T cell staining, rabbit anti-mouse CD3 antibody (abcam, Cambridge, UK; Cat # ab21703) was used in combination with Vector Blue development (Vector Blue Alkaline Phosphatase Substrate Kit, Vector Laboratories; Cat # SK-5300). Immunohistochemical staining was performed prior to laser capture microdissection to identify B cell aggregates in MP4-immunized mice and typical T cell infiltrates in the MOG:35–55 model.

### LCM and RNA isolation

Before starting the analysis, a 1% cresyl violet acetate solution was prepared by dissolving cresyl violet acetate (Sigma-Aldrich; Cat # C5042) in 50% ethanol. Subsequently, the prepared FrameSlides (Leica) were placed in 70% ethanol for 2 min. After staining the samples for 30 s with 1% cresyl violet acetate solution, the slides were shortly dipped in 70% ethanol followed by 100% ethanol. The slides were dried at room temperature for 2 min prior to using the microscope. To perform LCM, the LCM microscope and laser system (Leica LMD700) of the Department of Food Chemistry of the University of Würzburg was used. The target structures were cut out of the stained tissue by the laser. The dissected tissue was captured in RNase-free tubes and kept on dry ice. For isolation of RNA, RNeasy® Micro Kit (Qiagen, Hilden, Germany; Cat # 74004) was used according to the manufacturer’s instructions. RNA samples were stored at − 80 °C.

### Gene sequencing

The following procedure was performed by the Core Unit Systems Medicine (CU SysMed) at the Medical Faculty of the University of Würzburg. Bioanalyzer 2100 (Agilent Technologies, Santa Clara, CA, USA) was used to test RNA quantity and quality. Libraries for RNA sequencing were prepared from 8–28 ng of total RNA. After purification of poly-A RNA from each sample, it was converted to cDNA and linked to Illumina adapters by using the Illumina TruSeq stranded mRNA Kit following the manufacturer’s instructions (Illumina, San Diego, CA, USA). Subsequently, samples were multiplexed and the sequencing was performed on an Illumina NextSeq 500 in a 75-nt single-end setting using a high-output run mode. The generated raw reads were processed using FastQC 0.11.6 for assessing read quality, amount of duplicates, and presence of adapter sequences. After this, the Illumina TruSeq adaptors were cleaved using cutadapt (version 1.16) and resulting reads were further trimmed keeping a quality drop value below a mean of Q20. Further, the processed sequences were mapped to the mouse genome using the short read aligner STAR (version-2.5.2b) with genome and annotation files retrieved from GENCODE (July 2017, GRCm38.p5, M16). For all the studied samples, the proportion of reads mapped to the mouse reference genome ranged between 81% and 83% in total. The sequences aligning to specific genes were quantified using bedtools subcommand intersect (version 2.15.0). Next, the differentially expressed genes were identified using DESeq2 (version 1.16.1). Only the genes having a Benjamini-Hochberg corrected *p* value below 0.05 were classified as significantly differentially expressed (DEGs). The data were visualized as MA plot using DESeq2’s function plotMA. To compare the groups, heatmaps were used to represent genes having *p* adjusted values below 0.05 and an absolute log_2_ fold-change equal or above 2. The RNA sequencing data presented in this work have been deposited at the NCBI Gene Expression Omnibus and can be accessed through GEO series accession number GSE GSE125144 (https://www.ncbi.nlm.nih.gov/geo/query/acc.cgi?acc=GSE125144).

### Statistical analysis

Statistical analysis was used to determine the significance between the different amounts of T_H_17 cells in the different groups of mice. Statistical analysis and preprocessing of the data were performed using SciPy (1.1.0), StatsModels (0.9.0), scikit-learn (0.19.1), and the imbalanced-learn package (0.3) with Python 3.6.6. The data of the different groups were first upsampled to obtain at least four samples via the “resample” method from scikit-learn, which is the first step of the bootstrapping method. From this point on, the data of the corresponding groups were upsampled via the synthetic minority oversampling technique (SMOTE class from the imbalanced-learn package). Interpolated newly generated samples were obtained until the number of samples was matched from which the pooling of the data originated (10 for all groups except 11 for the mice with chronic EAE). The Shapiro-Wilk test revealed that the data were not drawn from a normal distribution. The consecutively performed Kruskal-Wallis *H* test for independent samples as a non-parametric test was then applied followed by the computation of the Tukey Honest Significant Differences (TukeyHSD). *p* values < 0.05 were considered as significant.

## Results

### LTi cells are absent in the cerebellum of EAE mice

**Fig. 3:**
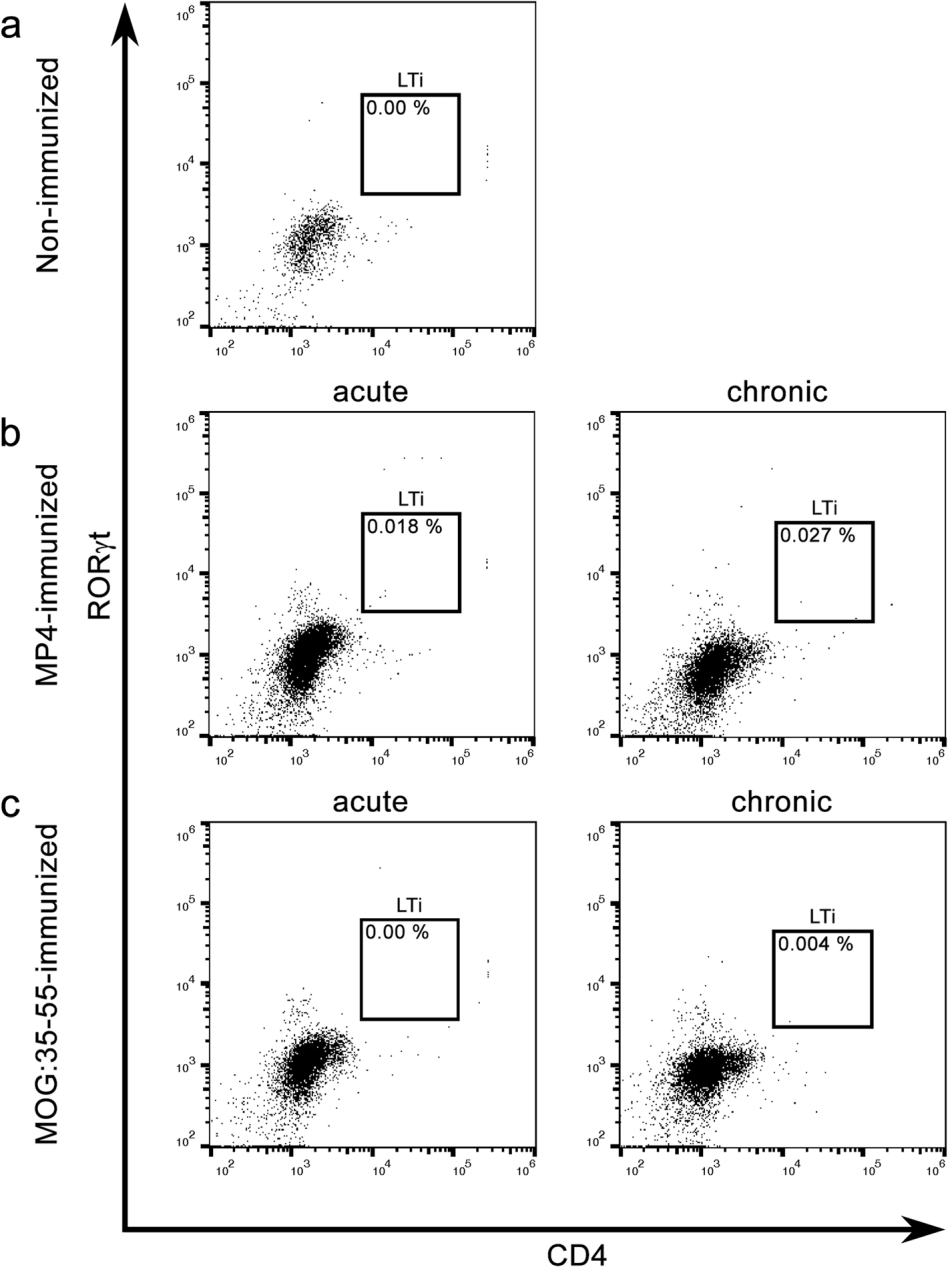
Flow cytometry data for LTi cell identification. The experiments were performed in non-immunized control and different groups of EAE mice. Cells were gated to detect CD3^−^CD5^−^CD4^+^RORγt^+^ LTi cells in the cerebellum. **a** Non-immunized mice were compared to **b** MP4-immunized EAE mice in the acute and chronic stage of disease and to **c** MOG:35–55-immunized EAE mice in the acute and chronic stage of disease

**Fig. 4:**
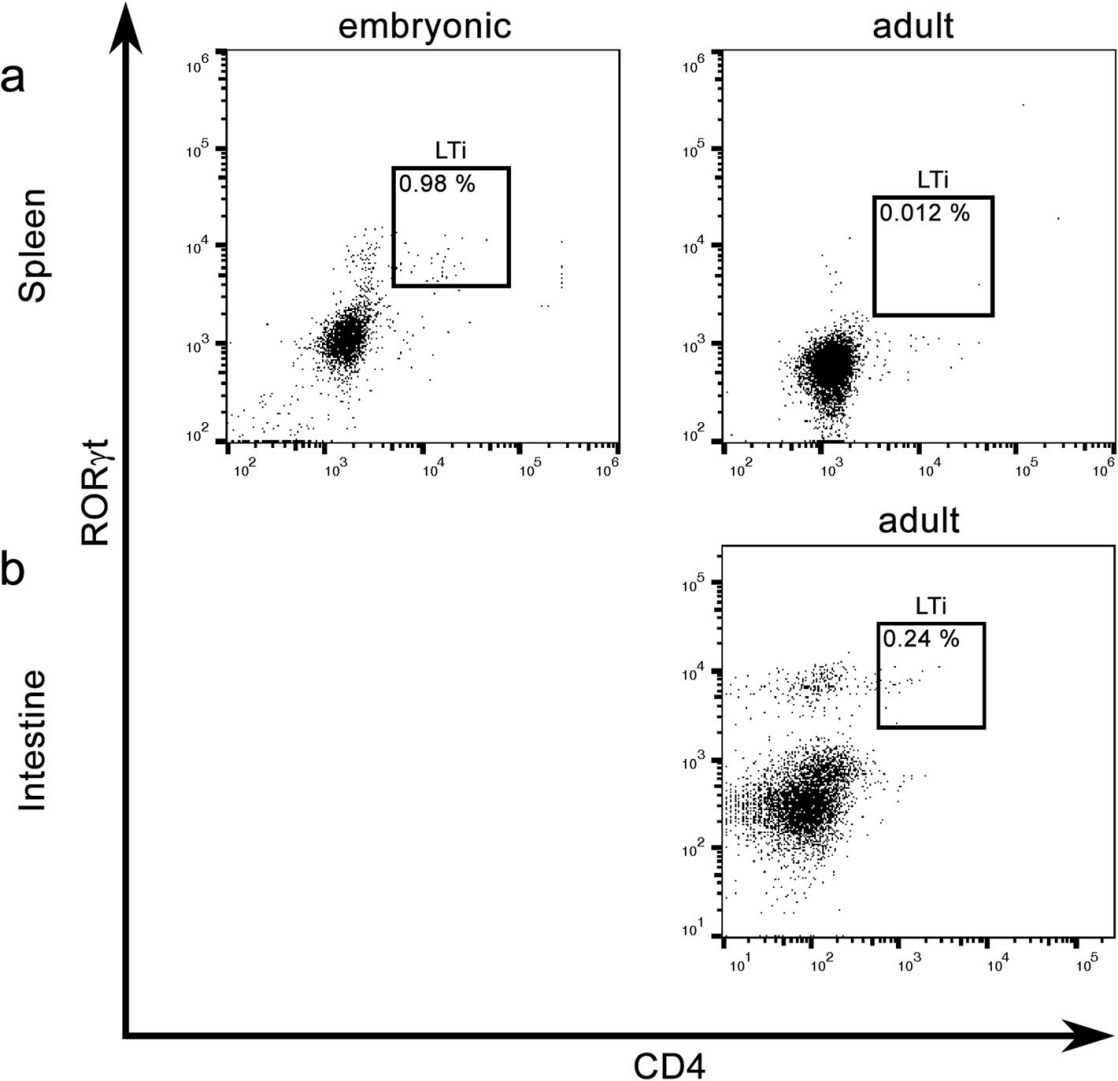
Flow cytometry data for LTi cell identification in mouse embryonic spleen and adult intestinal tissue. Comparison between **a** embryonic and adult spleen cells. CD3^−^CD5^−^CD4^+^RORγt^+^ LTi cells of splenic tissue are shown in the gates. **b** Flow cytometry analysis of intestinal tissue. CD3^−^CD5^−^CD4^+^RORγt^+^ LTi cells are shown in the gate

For flow cytometry experiments of the cerebellum and spleen, ten mice in each group (11 for the chronic stage of MP4-induced EAE) were divided into two cohorts and the tissues for each cohort were pooled. MP4-immunized B6 mice were analyzed either at the peak of disease (*n* = 2 × 5) or at the chronic stage of disease (*n* = 1 × 5 and 1 × 6) by flow cytometry. For analyzing mice during acute EAE, the animals were culled 16.00 ± 0.33 days after immunization with a mean score of 2.55 ± 0.20. To study the chronic stage of EAE, we analyzed mice with a mean score of 2.48 ± 0.08 at 72.10 ± 0.31 days after immunization. Furthermore, the same experiments were performed for MOG:35–55-immunized B6 mice during acute (*n* = 2 × 5) and chronic EAE (*n* = 2 × 5). These mice were culled 13.50 ± 0.50 days after immunization showing a mean score of 2.58 ± 0.20 and after 54 days with a mean score of 2.70 ± 0.06. Non-immunized mice (*n* = 2 × 5) served as controls. For analyzing the infiltrating immune cells within the CNS, we focused on the cerebellum, because in previous studies, we could confirm the presence of infiltrates in the cerebellum of both mouse models and the preferential formation of B cell aggregates in MP4-induced EAE in this brain region [10, 11]. As shown in Fig. 3 and Table 3, no significant amount of CD3^−^CD5^−^CD4^+^RORγt^+^ LTi cells could be detected in the cerebella of control and EAE mice. In addition, LTi cells were absent in the spleens of all three groups (Table 4). To confirm the functionality of our staining process and gating strategy, we measured intestinal cells of adult mice and spleen cells of embryos (Fig. 4). We detected a small amount of 0.24% ± 0.06 LTi cells in the intestine of *n* = 2 mice. To perform analysis of embryonic tissue, 16 embryonic spleens were pooled of two pregnant mice. While the spleens of the adult mice showed no presence of LTi cells, embryonic tissue possessed a slightly increased number of 0.98% of the desired cell population.

**Table 3:** Percentage of LTi, T_H_17 cells, and ILCs in the cerebellum of MP4-immunized and control mice

Mouse model	% of LTi cells	% of T_H_17 cells	% of ILCs
Non-immunized B6 mice			
*n* = 2 × 5	0.000 ± 0.000	0.00 ± 0.00	0.22 ± 0.01
MP4-immunized mice at acute stage of disease			
*n* = 2 × 5	0.018 ± 0.018	1.82 ± 0.10	0.67 ± 0.25
MP4-immunized mice at chronic stage of disease			
*n* = 1 × 5 and 1 × 6	0.027 ± 0.006	5.66 ± 0.22	0.24 ± 0.02
MOG:35–55-immunized mice at acute stage of disease			
*n* = 2 × 5	0.000 ± 0.000	2.82 ± 0.18	0.87 ± 0.35
MOG:35–55-immunized mice at chronic stage of disease			
*n* = 2 × 5	0.004 ± 0.001	3.16 ± 0.02	0.67 ± 0.12

The values relate to the percentage of living lymphocytes. The mean of the values ± SEM is shown

**Table 4:** Percentage of LTi and T_H_17 cell in the spleens of MP4-immunized and control mice

Mouse model	% of LTi cells	% of T_H_17 cells
Non-immunized B6 mice		
*n* = 2 × 5	0.012 ± 0.005	0.155 ± 0.005
MP4-immunized mice at acute stage of disease		
*n* = 2 × 5	0.013 ± 0.007	0.550 ± 0.100
MP4-immunized mice at chronic stage of disease		
*n* = 1 × 5 and 1 × 6	0.009 ± 0.002	0.435 ± 0.045
MOG:35–55-immunized mice at acute stage of disease		
*n* = 2 × 5	0.003 ± 0.003	0.565 ± 0.105
MOG:35–55-immunized mice at chronic stage of disease		
*n* = 2 × 5	0.019 ± 0.000	0.375 ± 0.005

The values relate to the percentage of living lymphocytes. The mean of the values ± SEM is shown

### ILCs are present in the cerebellum of EAE mice

**Fig. 5:**
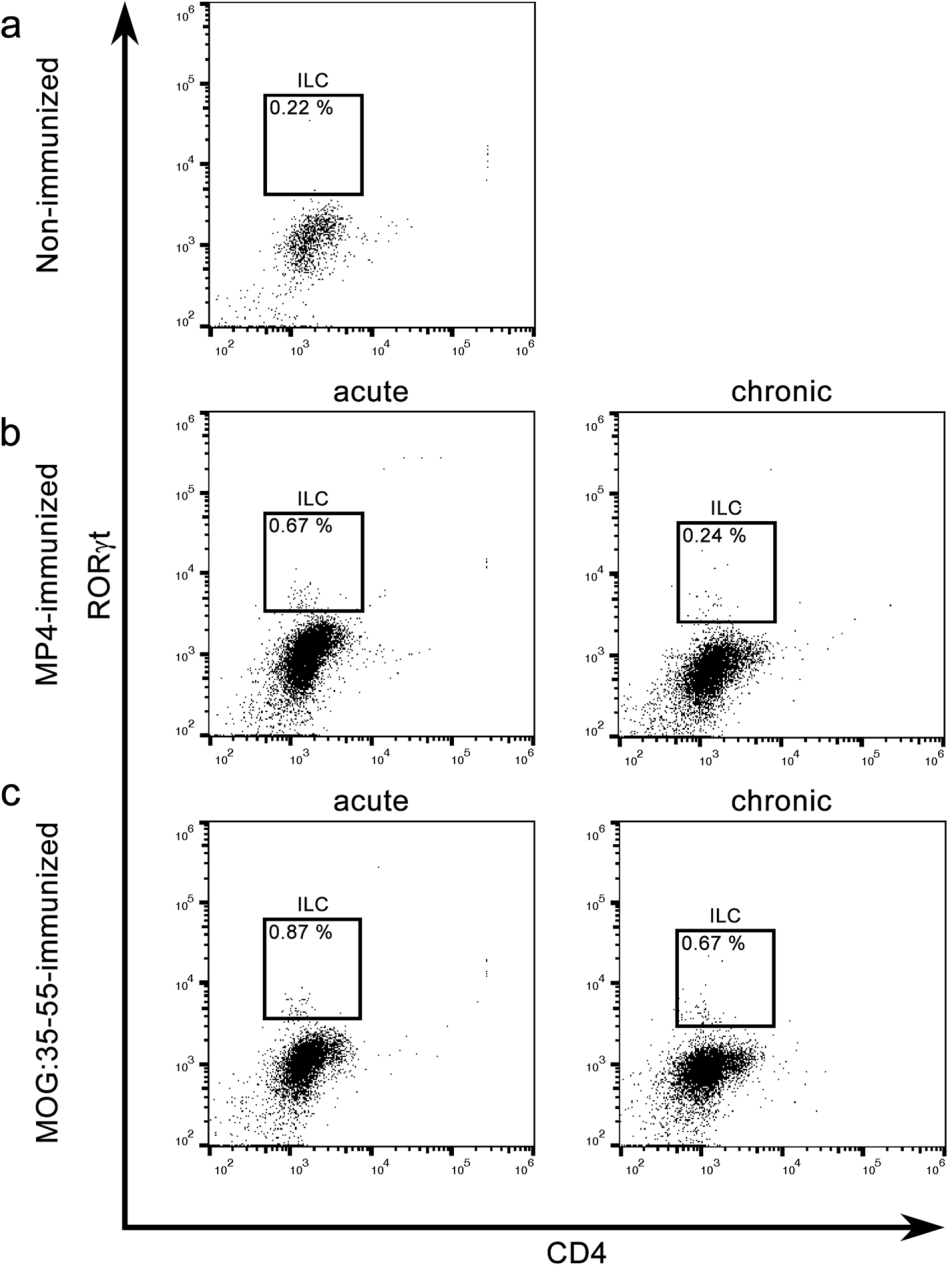
Flow cytometry data for CD4^-^ ILC identification. The same mouse groups as for LTi cell identification were used. The gates represent CD3^−^CD5^−^CD4^−^RORγt^+^ ILCs in the cerebellum. The figure shows the comparison between **a** non-immunized, **b** MP4-immunized mice in the acute and chronic stage of the disease and **c** MOG:35–55-immunized mice in the acute and chronic stage of the disease

We also determined whether ILCs were present in the cerebellum of EAE mice. We used the same groups of mice as for LTi cell analysis and followed the gating strategy shown in Fig. 2. ILCs were characterized as CD3^−^CD5^−^CD4^−^RORγt^+^. In contrast to LTi cells, we detected a small number of ILCs, especially in the acute stage of the disease in the MP4 model and at both time points in MOG:35–55-immunized mice (Fig. 5, Table 3).

### T_H_17 cells infiltrate the cerebellum of EAE mice

**Fig. 6:**
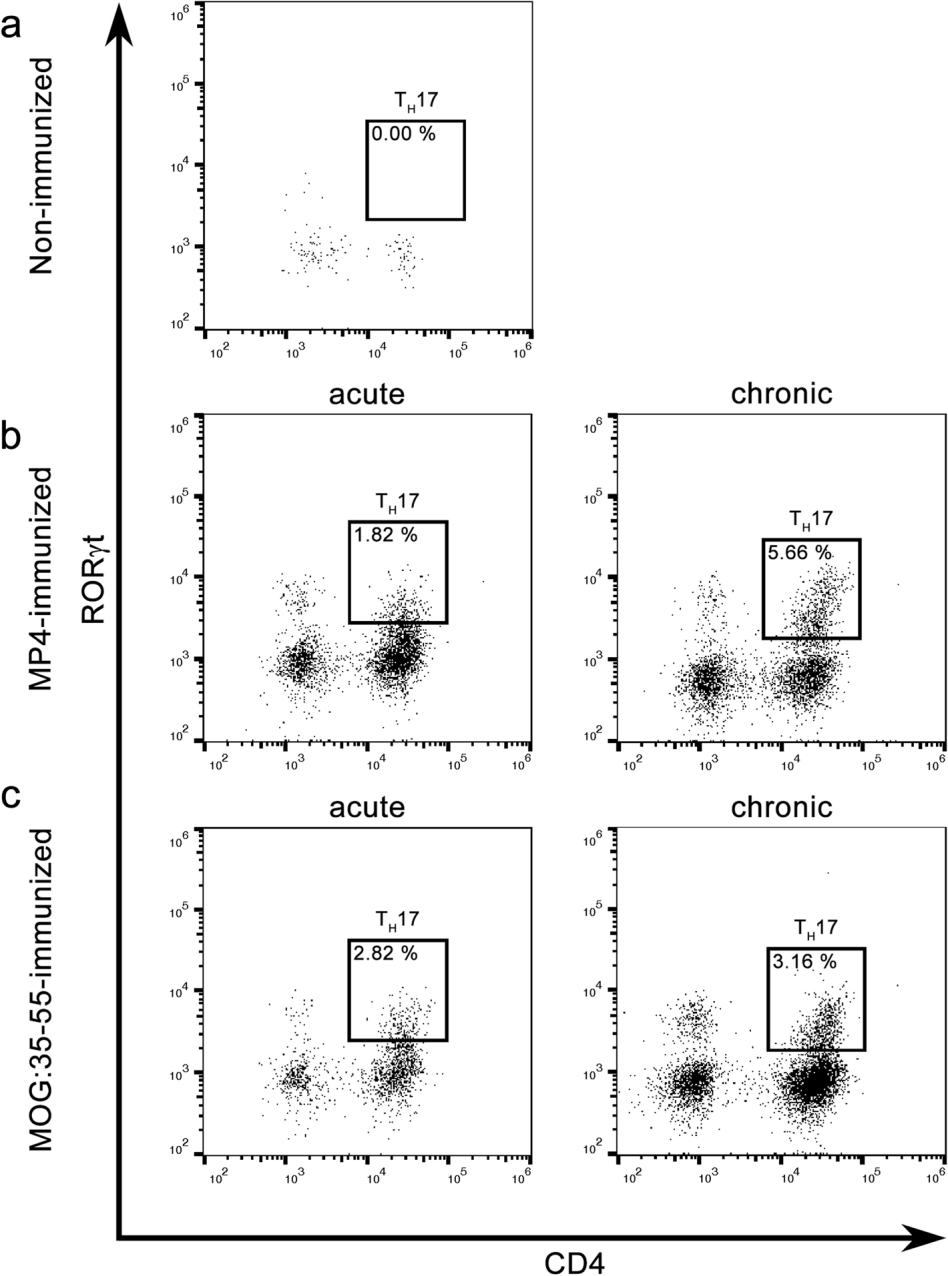
Flow cytometry data for T_H_17 cell identification. The same groups of mice were analyzed as for LTi cell identification. The gates show CD3^+^CD5^+^CD4^+^RORγt^+^ T_H_17 cells in the cerebellum. The comparison between **a** non-immunized, **b** MP4-immunized mice during acute and chronic EAE and **c** MOG:35–55-immunized mice during acute and chronic EAE is shown

For T_H_17 identification, we analyzed the same mice as above but used another gating strategy (Fig. 2). Figure 6 and Table 3 demonstrate that CD3^+^CD5^+^CD4^+^RORγt^+^ T_H_17 cells were present in the cerebellum of all EAE mice. Moreover, we observed an increase of this cell population during the course of MP4-induced EAE. Compared to 1.82% ± 0.10 T_H_17 cells detected at the peak of disease, the chronic cohorts of MP4-immunized B6 mice showed a significant increase to 5.66% ± 0.22 T_H_17 cells (*p* < 0.05). Only a slight increase was observed in MOG:35–55-induced EAE. Here, we detected 2.82 ± 0.18% of T_H_17 cells in the acute stage of the disease and 3.16 ± 0.02% of T_H_17 cells in the chronic stage. In non-immunized B6 mice, T_H_17 cell infiltration into the cerebellum was not observed. Compared to the cerebellum, only a small number of T_H_17 cells could be detected in the spleens of MP4- and MOG:35–55-immunized mice (Table 4).

### Characterization of gene expression in B cell aggregates of MP4-immunized mice

**Fig. 7:**
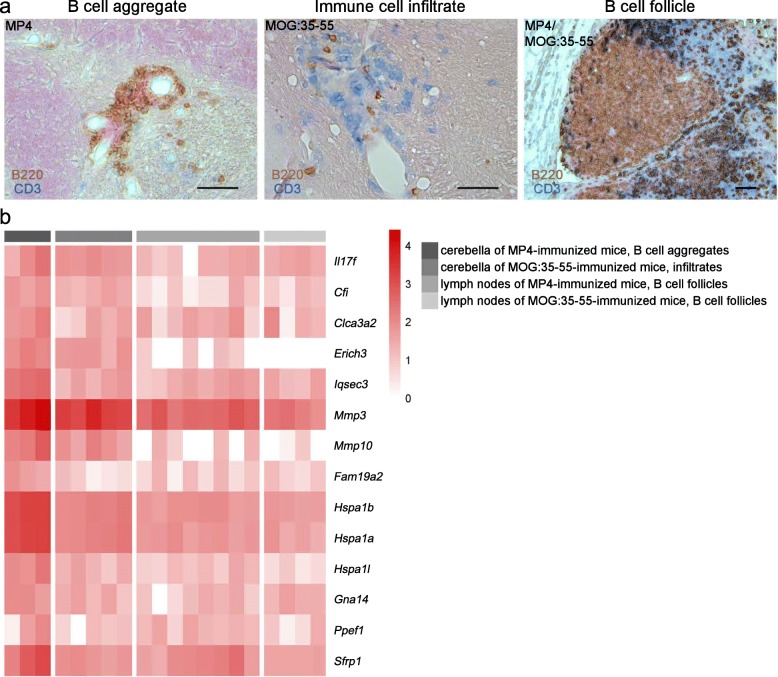
RNA sequencing analysis of B cell aggregates. **a** Dissected tissue comprised B cell aggregates from the cerebellum of MP4-immunized mice, diffuse immune cell infiltrates from the cerebellum of MOG:35–55-immunized mice and isolated B cell follicles from the draining lymph nodes of both models. Scale bars represent 50 μm. **b** Data of the different kinds of dissectates were compared using a heatmap. All included genes showed a log_2_ fold increase equal or above 2 and a *p* value below 0.05

MP4-immunized mice were dissected 58.44 ± 1.76 days after immunization showing a mean score of 2.22 ± 0.22. Mice suffering from MOG:35–55-induced EAE were culled 13 days after immunization with a mean score of 2.70 ± 0.05. While B cell aggregates are generally not observed in the MOG:35–55 model [11], MP4-immunized mice were kept until the chronic stage of the disease to ensure that B cell aggregates had developed, which were defined as tight perivascular clusters of more than 20 B cells. Using LCM, immune cell infiltrates were isolated from each respective tissue. Gene expression profiles of B cell aggregates that had been isolated from the cerebellum of mice with MP4-induced EAE were compared to immune cell infiltrates of MOG:35–55-immunized mice, which mainly consisted of T cells, and to B cell follicles from the draining lymph nodes derived from both models. Genes showing a log_2_ fold-increase equal or above 2 and a *p* value below 0.05 in B cell aggregates compared to the other samples were selected and summarized using a heatmap (Fig. 7). Our results show a significant upregulation of genes of different families in B cell aggregates of MP4-immunized mice compared to the controls. Besides *Il17f*, we detected two members of the matrix metalloproteinase (*Mmp*) gene family, i.e., *Mmp3* and *Mmp10*; heat shock protein (*Hsp*) genes of the *Hsp70* family, i.e. *Hspa1a*, *Hspa1b*, and heat shock protein 1-like (*Hspa1l*); and family with sequence similarity 19, member A2 (*Fam19a2*). Moreover, the complement component factor i (*Cfi*) and the chloride channel accessory 3A2 (*Clca3a2*) gene were upregulated. Further genes were glutamate rich 3 (*Erich3*), IQ motif and Sec7 domain 3 (*Iqsec3*), guanine nucleotide binding protein, alpha 14 (*Gna14*), protein phosphatase with EF hand calcium-binding domain 1 (*Ppef1*), and secreted frizzled-related protein 1 (*Sfrp1*).

## Discussion

The aim of our study was to investigate key mechanisms and molecules involved in the formation of B cell aggregates in the CNS of MP4-immunized mice. A particular focus was set on the role of LTi cells. Some studies have already provided evidence for the involvement of LTis in the formation of TLOs. Meier and colleagues have shown that an overexpression of interleukin-7 (IL-7) in transgenic mice induced the accumulation of LTi cells and the development of new Peyer’s patches, ectopic lymph nodes, and cecal patches [22]. Further experiments have demonstrated the formation of intestinal lymphoid tissue after transfer of LTi cells into CXCR5^−/−^ newborn mice [23]. Besides LTi cells, T_H_17 cells also seem to play an important role in TLO development. The transfer of MOG-specific T_H_17 cells led to the formation of ectopic lymphoid tissue in the CNS of mice. IL-17 and the T_H_17 cell surface molecule podoplanin have initially been suggested to be involved in this process [25], while further investigation has demonstrated that podoplanin also has an inhibitory effect on T cells [29]. Hence, podoplanin might play a dual role in TLO development, which needs to be further investigated. Here, we set out to identify the roles of LTi cells in the CNS of mice suffering from EAE, in particular in the MP4 model, which is characterized by ectopic lymphoid tissue formation in the chronic stage of the disease [12]. B cell aggregates were also described in patients suffering from secondary progressive MS and were associated with more rapid disease progression and cortical histopathology [3, 4]. Although MP4-immunized mice do not display disease progression so that B cell aggregate formation cannot be correlated to clinical disease parameters, MP4-induced EAE represents a convenient model to study key molecules and mechanisms involved in the development of ectopic lymphoid organs [12]. Our data demonstrate the absence of LTi cells in the cerebella of MP4- and MOG:35–55-immunized mice throughout the disease course. While LTi cells have been shown to induce the development of ectopic lymphoid tissue in the periphery of mice [22, 23], our results provide no evidence of a key role of this cell population in the CNS of EAE mice. As positive control, we analyzed the presence of LTi cells in the embryonic spleen. LTi cells migrate from the liver to the target tissue, where they induce the development of SLOs [21]. Therefore, LTi cells can be detected in the spleen during embryogenesis [30]. For adult mice, we used the intestine as a positive control. Studies have shown that LTi cells persisted in the intestine and were important to support the innate immune system [31, 32]. Along these lines, we detected a low number of LTi cells both in the embryonic spleen and in the intestine of adult mice. In humans, the occurrence of CD3^−^RORγt^+^ ILCs has been described in the CNS of a subgroup of MS patients in association with B cell follicle-like structures [24]. According to our definition of LTi cells, being described as CD3^-^CD5^−^CD4^+^RORγt^+^, the results of Serafini and colleagues are not in conflict with our data because of no further characterization of CD3^−^RORγt^+^ cells in their paper [24]. Nevertheless, we cannot exclude that few LTi cells still migrate into the CNS of MP4-immunized mice or are present at an earlier time point to induce lymphoid tissue formation. Detection of small cell populations always implicates difficulties. LTi cells represented a minority of the total leukocyte population in our control tissues and, therefore, it might be hard to detect a possibly even smaller amount in the cerebellum. Similar to the paper of Serafini, we also detected CD3^−^RORγt^+^ cells in the cerebellum of EAE mice, which we defined as CD3^−^CD5^−^CD4^−^RORγt^+^ ILCs. Overall, the numbers detected in our study were quite low. Only a slight increase could be observed during the acute stage of MP4-induced EAE and in the MOG:35–55 model. While B cell aggregates are a hallmark of the chronic stage of MP4-induced EAE, these structures do not occur in MOG:35–55-immunized mice [11]. In particular, chronic MP4-immunized mice showed almost no ILCs. Moreover, the amount of ILCs, which were detected in the acute stage of MP4-induced EAE, was comparable to the numbers of MOG:35–55-immunized mice. Therefore, it remains unclear if ILCs play any important role in ectopic lymphoid tissue formation in the CNS. Next to LTi cells and ILCs, we focused on the presence of CD3^+^CD5^+^CD4^+^RORγt^+^ T_H_17 cells in the cerebellum of EAE mice. According to the human study, which showed a much higher frequency of CD3^+^RORγt^+^ than of CD3^−^RORγt^+^ cells [24], we also detected CD3^+^CD5^+^CD4^+^RORγt^+^ T_H_17 cells in the CNS of both mouse models. Sharing a lot of common markers with LTi cells [27], it becomes obvious that T_H_17 cells could possibly undertake the tasks of LTi cells in the process of lymphoid tissue formation. Yet, they may not be the only decisive factor. On the one hand, one would expect the highest number of T_H_17 cells in the acute MP4 cerebellum. On the other hand, although the MOG:35–55 model is characterized by lack of B cell aggregates in the CNS [11], the frequencies of T_H_17 cells were comparable between the MP4 and MOG:35–55 model during the acute stage of the disease. Only a slight increase in the number of T_H_17 cells was observed in chronic MP4-immunized mice, which may also be attributed to the gating strategy. Overall, the formation of B cell aggregates and their evolution into ectopic lymphoid structures is certainly a complex process that involves more than one cell type and molecule. Indeed, when performing RNA sequencing on dissected B cell aggregates from chronic MP4-EAE mice, we detected a range of upregulated molecules compared to SLOs and to the MOG:35–55 model. In particular, we detected a significantly higher expression of *Il17f*, which is characteristic of T_H_17 cells [33]. Moreover, B cell aggregates expressed two members of the *Mmp* gene family, i.e., *Mmp3* and *Mmp10*. On the one hand, MMP-3 has been shown to assert neuroprotective function, e.g., by reducing Fas/FasL-mediated apoptosis [34]. On the other hand, MMP-3 could be involved in the pathophysiology of MS by degrading the blood brain barrier [35]. Unlike MMP-3, MMP-10 has not been mentioned in association with MS before. Yet, MMP-10 has been described to play a role in autoimmune processes in synovial pathology [36]. Furthermore, HSPs were highly expressed in MP4-related B cell aggregates. It is a moot question, whether heat shock proteins of the HSP70 family play a negative or beneficial role in MS pathogenesis, because of their contradictory function. Besides a neuroprotective role, HSP70 acts as an immunomodulator, for example by triggering the expression of pro-inflammatory cytokines [37, 38]. Moreover, it has been suggested that FAM19A2 has an immunomodulatory function, acts as a neurokine, and might be involved in axonal sprouting [39]. In addition, RNA sequencing showed further genes to be upregulated in B cell aggregates of the MP4 model. Although all of these genes have not been described in association with neurological diseases and autoimmunity before and there are currently no reports that they are involved in the process of ectopic tissue formation, they should not be excluded from further investigation.

## Conclusion

This study focused on the mechanisms and molecules, which could be involved in ectopic lymphoid tissue formation in the CNS. LTi cells, the initiators of SLO development, did not seem to play an important role in the investigated process. Furthermore, only a small amount of ILCs was present in the CNS. Corresponding to a previously postulated role of T_H_17 cells in B cell aggregate formation, we detected such cells in the CNS of MP4-immunized mice. In addition, we observed the upregulation of specific genes in association with B cell aggregates in the cerebellum of MP4-immunized mice. Whether and how each of these genes and their products are involved in the development of ectopic lymphoid organs remains to be shown.

## Abbreviations

B6: C57BL/6
CFA: Complete Freund’s adjuvant
Cfi: Complement component factor i
Clca3a2: Chloride channel accessory 3A2
CNS: Central nervous system
DAB: Diaminobenzidine
DDT: Dithiothreitol
EAE: Experimental autoimmune encephalomyelitis
Erich3: Glutamate rich 3
FACS: Fluorescence-activated cell sorting
Fam19a2: Family with sequence similarity 19, member A2
FBS: Fetal bovine serum
FSC: Forward scatter
FSC-A: Forward scatter area
FSC-H: Forward scatter height
FVS450: Fixable viability stain 450
Gna14: Guanine nucleotide binding protein, alpha 14
HBSS: Hank’s balanced salt solution
Hsp: Heat shock protein
Hspa1l: Heat shock protein 1-like
IFA: Incomplete Freund’s adjuvant
IHC: Immunohistochemistry
IL: Interleukin
ILC: Innate lymphoid cells
Iqsec3: IQ motif and Sec7 domain 3
LCM: Laser capture microdissection
LTi: Lymphoid tissue inducer cells
MBP: Myelin basic protein
Mmp: Matrix metalloproteinase
MOG: Myelin oligodendrocyte glycoprotein
MP4: MBP-PLP fusion protein
MS: Multiple sclerosis
NGS: Normal goat serum
PBS: Phosphate-buffered saline
PET: Polyethylene terephthalate
PFA: Paraformaldehyde
PLP: Proteolipid protein
Ppef1: Protein phosphatase with EF hand calcium-binding domain 1
SEM: Standard error of the mean
Sfrp1: Secreted frizzled-related protein 1
SLO: Secondary lymphoid organ
SP-MS: Secondary progressive MS
SSC: Sideward scatter
SSC-A: Sideward scatter area
TLO: Tertiary lymphoid organ
